# Trends in Pneumonia Mortality Rates and Hospitalizations by Organism, United States, 2002–2011^1^

**DOI:** 10.3201/eid2209.150680

**Published:** 2016-09

**Authors:** Brandon A. Wuerth, John P. Bonnewell, Timothy L. Wiemken, Forest W. Arnold

**Affiliations:** University of Louisville, Louisville, Kentucky, USA

**Keywords:** pneumonia, case-fatality rate, influenza, Streptococcus pneumoniae, Staphylococcus aureus, hospitalization, respiratory infections, bacteria, staphylococci, streptococci

## Abstract

Because the epidemiology of pneumonia is changing, we performed an updated, population-based analysis of hospitalization and case-fatality rates for pneumonia patients in the United States. From 2002 to 2011, hospitalization rates decreased significantly for pneumonia caused by pneumococcus and *Haemophilus influenzae* but increased significantly for *Pseudomonas* spp., *Staphylococcus aureus*, and influenza virus.

Pneumonia is the leading cause of infection-related deaths in the United States, with potential for severe complications such as respiratory failure and sepsis (*1*). A recent nationwide study noted that prior studies may have overestimated a temporal reduction in mortality rate (*2*). As pneumonia epidemiology has changed, interest in following epidemiologic trends continues, particularly for the various etiologic organisms. Emergence of influenza A(H1N1)pdm09 virus has highlighted the role of influenza viruses as etiologic agents of pneumonia (*3*).

The epidemiology of pneumonia is constantly changing because of advances in preventive measures, diagnostic testing, and novel therapies. Although the epidemiology, by organism, of pneumonia in hospital patients was recently clarified (*4*), our study also considered the effects of influenza virus and included regional information. Our objective was to provide an updated population-based analysis of hospitalized pneumonia patients to determine the major etiologic agents and associated hospitalization rates, case-fatality rates, and patient demographic differences (age, sex, and region).

## The Study

In a retrospective cohort study, we examined hospitalizations of pneumonia patients using discharge data from the publicly available Nationwide Inpatient Sample (NIS) database (*5*). The University of Louisville Institutional Review Board did not require a review because the project did not meet the common rule definition of human subjects’ research. We completed Healthcare Cost and Utilization Project Data Use Agreement Training. 

The study included patients >18 years of age discharged with a principal diagnosis of pneumonia according to standards of the International Classification of Disease, Ninth Revision, Clinical Modification (ICD-9-CM). We also included those with a principal diagnosis of sepsis or respiratory failure and a secondary diagnosis of pneumonia. Only ICD-9-CM codes for which the numbers of an organism were substantial were analyzed: pneumococci, *Klebsiella* spp., *Pseudomonas* spp*.*, *Haemophilus influenzae*, *Staphylococcus aureus*, and influenza virus. We followed US Census Bureau definitions in classifying cases by US region (*6*).

The NIS database contains an ≈20% stratified sample of US community hospitals. From that sample, the Agency for Healthcare Research and Quality calculated national estimates by weighting each discharge on the basis of hospital location, teaching status, bed number, and ownership control, relative to all US hospitals, which permitted us to estimate the number of US hospitalizations. For each patient discharged, we recorded age, sex, region, principal and secondary diagnoses (up to 15), and whether patient had died from any cause. Population estimates were obtained from the US Census Bureau (*7*).

Primary outcomes were temporal trends in hospitalization and case-fatality rates for pneumonia based on infecting organism as determined by ICD-9 coding. We then calculated the rate for hospitalizations caused by each organism using the number of hospitalizations from the NIS database as the numerator and US Census Bureau population estimates as the denominator. Discharge codes do not specify where the pneumonia was acquired, and although hospitalization rate was used, this does not necessarily indicate community-acquired cases. We calculated the all-cause case-fatality rate for each organism similarly, using the number of deaths.

We extracted data from the NIS using SAS version 9.3 (SAS Institute, Inc., Cary, NC, USA) and calculated adjusted odds ratios for age ranges, sex, and region using multivariate logistic regression. Study period differences were determined with the online *z* test calculator of the Healthcare Cost and Utilization Project (p<0.05 was significant) (*8*). Other descriptive statistics were completed with Excel 2013 (Microsoft Corp., Redmond, WA, USA).

*S. aureus* was the most commonly identified organism that caused pneumonia, at 19.2 cases/100,000 population (Table 1). Pneumococcus and influenza virus were more frequent causes in the Midwest, *Klebsiella* and *Pseudomonas* spp. were more frequent causes in the South, and *H. influenzae* and *S. aureus* were more frequent agents in the West.

**Table 1:** Hospitalization rates (hospitalization/100,000 population) for 6 causative agents, by sex and region, United States, 2002–2011

Category	Rate (adjusted odds ratio)					
	Pneumococcus, n = 379,400	*Klebsiella* spp., n = 109,515	*Pseudomonas* spp., n = 330,302	*Haemophilus influenzae*, n = 80,281	*Staphylococcus aureus*, n = 575,573	Influenza virus, n = 270,237
Overall	12.6	3.6	11.0	2.7	19.2	9.0
Sex						
M	13.0 (1)	4.1 (1)	12.3 (1)	2.7 (1)	20.5 (1)	8.0 (1)
F	12.3 (0.68)	3.2 (0.57)	9.7 (0.57)	2.6 (0.72)	17.9 (0.64)	10.0 (0.90)
Region						
Northeast	11.9 (1)	3.1 (1)	8.8 (1)	2.1 (1)	15.9 (1)	8.8 (1)
Midwest	15.1 (1.33)	3.1 (1.05)	11.5 (1.37)	3.3 (1.61)	17.7 (1.17)	12.8 (1.51)
South	12.8 (1.16)	4.6 (1.65)	13.0 (1.62)	2.8 (1.46)	22.5 (1.57)	9.0 (1.10)
West	10.7 (1.32)	3.0 (1.48)	9.1 (1.55)	2.3 (1.64)	17.9 (1.71)	5.5 (0.90)

**Figure 1:**
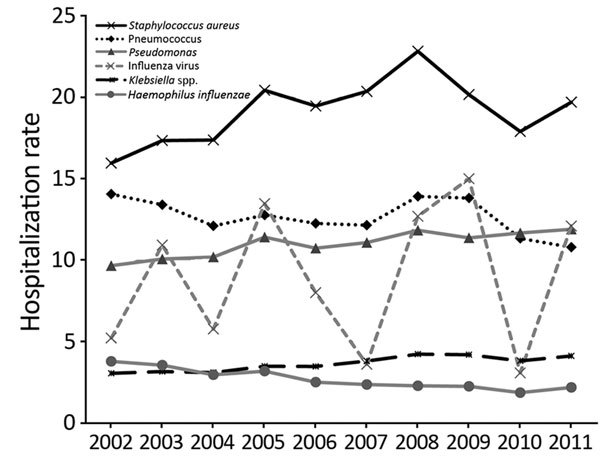
Hospitalization rates (hospitalizations/100,000 population) for patients with pneumonia for 6 causative agents, United States, 2002–2011.

Hospitalization rates, based on etiologic agent, changed during the study period (Figure 1). Overall, the rate for pneumonia hospitalizations caused by pneumococcus decreased 23% (p<0.001) and for *H. influenzae*, 42% (p<0.001). For *Klebsiella*, the rate increased 35% (p<0.001); for *Pseudomonas*, 23% (p<0.001); for *S. aureus*, 23% (p<0.001); and for influenza virus, 132% (p<0.001).

Case-fatality rate was highest for pneumonia caused by *S. aureus*: 15.6 deaths/100 cases (Table 2). Pneumococcal pneumonia, *Klebsiella*, *Pseudomonas*, and *S. aureus* were more frequent in the Northeast, whereas pneumonia caused by *H. influenzae* and influenza virus was more frequent in the West.

**Table 2:** All-cause case-fatality rate (deaths/100 cases for 6 causative agents, by sex and region, United States, 2002–2011

Category	Rate (adjusted odds ratio)

Pneumococcus, n = 25,112	*Klebsiella* spp., n = 15,687	*Pseudomonas* spp., n = 39,975	*Haemophilus influenzae*, n = 3,935	*Staphylococcus. aureus*, n = 89,540	Influenza virus, n = 9,417
Overall	6.6	14.3	12.1	4.9	15.6	3.5
Sex						
M	6.8 (1)	15.4 (1)	12.6 (1)	5.1 (1)	16.6 (1)	3.8 (1)
F	6.5 (0.91)	12.9 (0.81)	11.5 (0.87)	4.7 (0.86)	14.4 (0.83)	3.3 (0.84)
Region						
Northeast	8.1 (1)	20.5 (1)	15.3 (1)	5.8 (1)	18.9 (1)	3.9 (1)
Midwest	5.6 (0.67)	12.0 (0.53)	9.8 (0.62)	3.8 (0.64)	13.1 (0.67)	3.3 (0.83)
South	6.4 (0.82)	12.4 (0.56)	11.7 (0.75)	4.8 (0.86)	14.9 (0.78)	3.3 (0.92)
West	7.2 (0.91)	16.2 (0.76)	13.2 (0.85)	5.9 (1.06)	16.9 (0.89)	3.8 (1.07)

**Figure 2:**
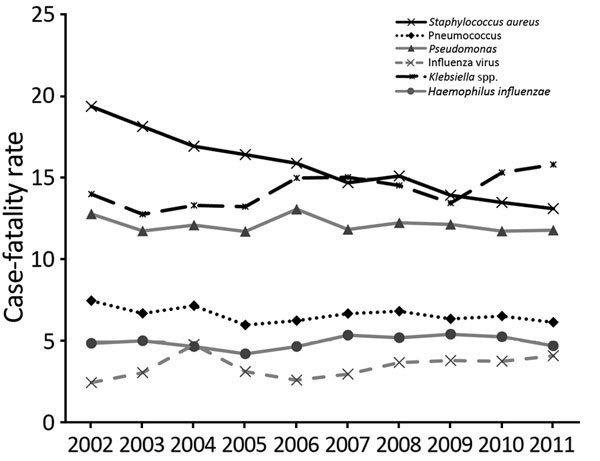
All-cause case-fatality rate (deaths/100 cases) for patients with pneumonia for 6 causative agents, United States, 2002–2011.

Overall case-fatality rates, based on causal agent (Figure 2), decreased for pneumococcus by 18% (p = 0.01), for *Pseudomonas* spp*.* by 8% (p = 0.29), for *H. influenzae* by 3% (p = 0.85), and for *S. aureus* by 32% (p<0.001). Rates increased for *Klebsiella* spp*.* by 13% (p = 0.26); and for influenza virus by 67% (p<0.001). Hospitalization and case-fatality rates by age are shown in Technical Appendix Tables 1, 2.

## Conclusions

We found that, from 2002 to 2011, the hospitalization rates for pneumonia caused by *Klebsiella* spp., *Pseudomonas* spp., *S. aureus*, and influenza virus increased, and those caused by pneumococcus and *H. influenzae* decreased. The case-fatality rate of influenza virus–caused pneumonia increased, while rates of pneumococcus-caused and *S. aureus*–caused pneumonia decreased. Hospitalization and case-fatality rates were higher for men (especially elderly) than for women.

Our results coincide with those of previous studies, which found that *S. aureus*, pneumococcus, and *Pseudomonas* spp. comprised a large percentage of all pneumonia cases (*9*,*10*). Our results also coincide with those of a similar study which used the NIS database during 1993–2011: a decrease in pneumonia caused by *Streptococcus* spp., *H. influenzae*, and *Pseudomonas* spp. and an increase in pneumonia caused by *Staphylococcus* spp. (*4*). However, our study also examined trends in pneumonia caused by *Klebsiella* spp. and influenza virus and regional variations. Another study that used similar methods found that adjusted odds ratio of an inpatient death from pneumonia decreased from 2002 to 2005 (*11*). Previous studies also found that male patients >75 years old carried the greatest disease prevalence and considerably greater hospitalization rates (185/10,000 population in 2006) (*12**,**13*).

US regional data are limited, and comparable results, based on clinical data, were not found. However, 2 sets of data from the Centers for Disease Control and Prevention (Atlanta, GA, USA) showed the highest incidence of pneumonia in the Midwest and the lowest in the West (*12*,*14*). Drivers of geographic differences could include variations in coding practices between regions and vaccination recommendation adherence (*15*).

Known changes in the epidemiology of pneumonia over the study period include the emergence of influenza A(H1N1)pdm09 virus, which caused 60.8 million cases of influenza and 12,469 deaths in 2009–2010 alone (*3*). We found that patients with influenza virus infections had the highest hospitalization rate during 2009. Many previous studies excluded influenza because it likely represents a separate clinical entity and the hospitalization rate can be difficult to interpret using calendar years (because early or late influenza seasons can skew yearly trends) (*2*,*11*).

Our study was limited by its reliance on ICD-9-CM coding to identify cases, potentially allowing misclassification. ICD-9-CM coding is based on discharge data, so determining whether the patient was diagnosed with pneumonia at admission or during hospitalization is not possible. Thus, we could not differentiate between community- or hospital-acquired cases. In addition, we did not examine unspecified cases of pneumonia that did not include an etiologic organism, the most commonly used pneumonia code. Decreases in hospitalization rates and case-fatality rates could be explained by improved outpatient management or increased rates of vaccination. Last, case-fatality rates were calculated by using deaths during the inpatient stay and not beyond, because coding did not permit 30-day outcomes.

In conclusion, knowing which etiologic agents are increasing or have the highest case-fatality rate is critical because treatment options and prognosis vary by organism, and resources must be allocated accordingly. Knowing patient demographic characteristics for each organism is also essential, to clarify the populations at greatest risk.

Technical Appendix Tables showing hospitalization rate per 100, 000 population by organism and age and all-cause case-fatality rate per 100 population by organism and age, United States, 2002–2011.

## References

[1] Heron M. Deaths: leading causes for 2010. Natl Vital Stat Rep. 2013;62:1–96. 24364902

[2] Lindenauer PK, Lagu T, Shieh MS, Pekow PS, Rothberg MB. Association of diagnostic coding with trends in hospitalizations and mortality of patients with pneumonia, 2003–2009. JAMA. 2012;307:1405–13. 10.1001/jama.2012.384 22474204

[3] Shrestha SS, Swerdlow DL, Borse RH, Prabhu VS, Finelli L, Atkins CY, Estimating the burden of 2009 pandemic influenza A (H1N1) in the United States (April 2009–April 2010). Clin Infect Dis. 2011;52(Suppl 1):S75–82 . 10.1093/cid/ciq012 21342903

[4] Smith SB, Ruhnke GW, Weiss CH, Waterer GW, Wunderink RG. Trends in pathogens among patients hospitalized for pneumonia from 1993 to 2010. JAMA Intern Med. 2014;174:1837–9 and. 10.1001/jamainternmed.2014.4344 PMC4329730 25200864

[5] Agency for Healthcare Research and Quality. Overview of the National (Nationwide) Inpatient Sample (NIS) [cited 2012 Nov 24]. https://www.hcup-us.ahrq.gov/nisoverview.jsp

[6] Henry J. Kaiser Family Foundation. Health coverage and care in the South in 2014 and beyond [cited 2014 Jun 19]. http://kff.org/disparities-policy/issue-brief/health-coverage-and-care-in-the-south-in-2014-and-beyond/

[7] US Census Bureau. Population estimates, historical data [cited 2012 Dec 20]. https://www.census.gov/popest/data/historical

[8] Z-test calculator [cited 2014 Jun 22]. http://hcupnet.ahrq.gov/ZTestCalc.jsp

[9] Micek ST, Kollef KE, Reichley RM, Roubinian N, Kollef MH. Health care-associated pneumonia and community-acquired pneumonia: a single-center experience. Antimicrob Agents Chemother. 2007;51:3568–73 . 10.1128/AAC.00851-07 17682100 PMC2043297

[10] DeFrances CJ, Lucas CA, Buie VC, Golosinskiy A. 2006 National Hospital Discharge Survey. Hyattsville (MD): National Center for Health Statistics; 2008. p. 1–20. 18841653

[11] Ruhnke GW, Coca Perraillon M, Cutler DM. Mortality reduction among pneumonia patients still substantial despite the impact of coding changes. Ann J Med. 2013;126:266–9. 10.1016/j.amjmed.2012.08.006 23410567

[12] Jokinen C, Heiskanen L, Juvonen H, Kallinen S, Kleemola M, Koskela M, Microbial etiology of community-acquired pneumonia in the adult population of 4 municipalities in eastern Finland. Clin Infect Dis. 2001;32:1141–54. 10.1086/319746 11283803

[13] Gutiérrez F, Masia M, Mirete C, Soldan B, Rodriguez JC, Padilla S, The influence of age and gender on the population-based incidence of community-acquired pneumonia caused by different microbial pathogens. J Infect. 2006;53:166–74. 10.1016/j.jinf.2005.11.006 16375972

[14] Song Y, Skinner J, Bynum J, Sutherland J, Wennberg JE, Fisher ES. Regional variations in diagnostic practices. N Engl J Med. 2010;363:45–53. 10.1056/NEJMsa0910881 20463332 PMC2924574

[15] Aronsky D, Haug PJ, Lagor C, Dean NC. Accuracy of administrative data for identifying patients with pneumonia. Am J Med Qual. 2005;20:319–28. 10.1177/1062860605280358 16280395

