# Supplementary material for: Trends in Pneumonia Mortality Rates and Hospitalizations by Organism, United States, 2002–2011
Source: Emerg Infect Dis. 2016 Sep;22(9):1624–7. doi: 10.3201/eid2209.150680 (PMC4994371; doi:10.3201/eid2209.150680)
Supplement: Technical Appendix — Tables showing hospitalization rate per 100, 000 population by organism and age and all-cause case-fatality rate per 100 population by organism and age, United States, 2002–2011. [file 15-0680-Techapp-s1.pdf]

Article DOI: http://dx.doi.org/10.3201/eid2209.150680

Trends in Pneumonia Mortality Rates and Hospitalizations by Organism, United States, 2002–2011

Technical Appendix

Technical Appendix Table 1. Hospitalization rate per 100 000 population by organism and age, (adjusted odds ratio, 95% CI); United States, 2002–2011

Age, y	Pneumococcus	*Klebsiella* spp,	*Pseudomonas* spp.	*Haemophilus influenzae*	*Staphylococcus aureus*	Influenza virus
18–24	2.1 (1)	0.3 (1)	4.5 (1)	0.2 (1)	2.9 (1)	3.9 (1)
25–34	3.8 (1.44, 1.40–1.48)	0.4 (1.14, 1.05–1.24)	3.3 (0.59, 0.57–0.60)	0.3 (1.15, 1.05–1.26)	3.6 (0.99, 0.97–1.02)	4.4 (0.88, 0.86–0.90)
35–44	7.2 (3.17, 3.09–3.26)	0.9 (2.92, 2.71–3.15)	3.4 (0.68, 0.66–0.70)	0.7 (2.84, 2.62–3.08)	5.9(1.88, 1.84–1.93)	5.3 (1.28, 1.25–1.31)
45–54	13.4 (4.64, 4.52–4.76)	2.7 (6.83, 6.37–7.33)	6.9 (1.05,1.03–1.07)	1.8 (5.62, 5.21–6.07)	13.3 (3.31, 3.24–3.39)	8.3 (1.62, 1.59–1.66)
55–64	21.7 (5.17, 5.04–5.30)	5.9 (10.21, 9.52–10.94)	16.7(1.74,1.71–1.78)	4.2 (9.05, 8.39–9.76)	27.1 (4.62, 4.52–4.73)	11.4 (1.54, 1.50–1.57)
65–74	38.9 (5.28, 5.15–5.42)	13.5 (13.29,12.40–14.24)	42.5 (2.53, 2.49–2.58)	10.2 (12.41, 11.51–13.37)	62.5 (6.08, 5.95–6.21)	21.7 (1.65, 1.62–1.69)
75–84	59.2 (5.17, 4.99–5.25)	22.3 (14.15, 13.20–15.16)	65.9 (2.55, 2.50–2.59)	16.9 (13.18, 12.2–14.2)	122.6 (7.69, 7.52–7.86)	45.5 (2.17, 2.13–2.22)
85–94	85.2 (5.49, 5.35–5.64)	31.5 (15.18, 14.15–16.27)	69.9 (2.06,2.02–2.10)	22.5 (13.01,12.06–14.03)	190.4 (9.03, 8.83–9.23)	82.1 (2.87, 2.81–2.93)
≥95	95.7 (7.19,6.92–7.47)	32.2 (18.52, 17.02–20.16)	46.8 (1.65, 1.58–1.73)	20.4(13.70,12.43–15.10)	171.1 (9.61, 9.32–9.91)	85.0 (3.37, 3.25–3.49)

Technical Appendix Table 2. All–cause case-fatality rate per 100 population by organism and age (adjusted odds ratio, 95% CI),United States, 2002–2011

Age, y	Pneumococcus	*Klebsiella* spp.	*Pseudomonas* spp.	*Haemophilus influenzae*	*Staphylococcus aureus*	Influenza virus
18–24	1.7 (1)	10.0 (1)	3.6 (1)	1.3 (1)	4.0 (1)	1.6 (1)
25–34	2.4 (1.48, 1.19–1.85)	9.0 (0.88, 0.67–1.18)	5.3 (1.51, 1.34–1.69)	1.5 (1.12, 0.51–2.43)	6.6 (1.73, 1.52–1.96)	2.2 (1.41, 1.18–1.69)
35–44	3.5 (2.13, 1.74–2.61)	8.8 (0.88, 0.68–1.13)	7.3 (2.08, 1.87–2.32)	3.2 (2.54, 1.29–4.99)	8.1 (2.15, 1.91–2.42)	1.8 (1.13 ,0.95–1.35)
45–54	4.9 (3.04, 2.49–3.70)	11.2 (1.14, 0.90–1.45)	10.3 (3.06, 2.77–3.38)	3.3 (2.57, 1.33–4.96)	10.4 (2.82, 2.52–3.15)	2.8 (1.83 ,1.56–2.14)
55–64	5.5 (3.47, 2.85–4.22)	12.2 (1.25, 0.99–1.58)	11.6 (3.51, 3.19–3.86)	3.5 (2.75, 1.44–5.29)	13.0 (3.64, 3.26–4.07)	3.0 (1.94 ,1.66–2.27)
65–74	6.7 (4.28, 3.52–5.21)	13.4 (1.39, 1.10–1.75)	12.9 (3.94, 3.59–4.33)	4.0 (3.20, 1.67–6.13)	15.3 (4.40, 3.95–4.91)	3.2 (2.08 ,1.78–2.43)
75–84	8.2 (5.34, 4.40–6.49)	16.4 (1.75, 1.39–2.20)	13.9 (4.30, 3.92–4.71)	6.0 (4.93, 2.58–9.42)	18.5 (5.56, 4.99–6.20)	3.8 (2.53 ,2.17–2.94)
85–94	11.6 (7.85, 6.45–9.55)	18.7 (2.06, 1.64–2.60)	15.8 (5.06, 4.60–5.56)	8.2 (6.94, 3.63–13.28)	20.5 (6.36, 5.70–7.10)	5.9 (4.03 ,3.46–4.69)
≥95	15.4 (11.05, 8.96–13.64)	20.0 (2.32, 1.79–3.01)	17.8(5.90, 5.12–6.81)	5.8 (4.86, 2.41–9.80)	21.8 (7.05, 6.25–7.95)	8.9 (6.46, 5.38–7.75)
